# Evaluating In Vivo Penetration of Yeast Rice Ferment Filtrate Into Skin Using Confocal Raman Microspectroscopy: A Pilot Study

**DOI:** 10.1111/srt.70166

**Published:** 2025-05-05

**Authors:** Fan Yang, Miao Guo, Hua Wang

**Affiliations:** ^1^ Department of Biotechnology College of Life Science and Technology Huazhong University of Science and Technology Wuhan China; ^2^ Research & Development Center Mageline Biology Tech Co., Ltd. Wuhan Hubei China

**Keywords:** confocal raman, delivery, rice ferment filtrate, skin barrier, skin penetration, spectroscopy

## Abstract

**Background:**

Investigating the skin penetration behavior of pharmaceuticals and cosmetics is crucial. Due to the Raman‐active properties of many active ingredients, Confocal Raman microspectroscopy (CRM) emerges as a promising, non‐invasive technique for penetration studies.

**Aim:**

To evaluate the in vivo penetration behavior of yeast rice ferment filtrate (RFF) into the skin using CRM.

**Methods:**

Raman spectra of RFF were acquired at 0.5, 1, 2, and 4 h post‐application using CRM. The penetration of RFF at various skin depths (10–40 µm) and different time points was analyzed. Multivariate analysis was applied to generate Raman spectral maps that can show RFF depth penetration and spatial distribution across different skin layers.

**Results:**

RFF surpassed the stratum corneum barrier within 30 min and penetrated into the dermis layer after 4 h of application, indicating rapid permeation kinetics. Quantitative analysis, based on Raman spectra, showed increasing RFF penetration over time, reaching 1.02%, 3.17%, 5.29%, and 8.75% after 0.5, 1, 2, and 4 h, respectively. The heat map visualized depth penetration and spatial distribution, indicating a significant increase in penetration with extended application durations.

**Conclusion:**

CRM proves to be a powerful tool for studying the in vivo penetration of RFF into the skin, offering valuable insights into the complex dynamics underlying its permeation behavior.

## 1 Introduction

The skin, as the body's largest organ, serves as an external barrier, shielding against external threats, regulating temperature, and facilitating metabolic processes [1]. Central to this barrier is the stratum corneum (SC), the outermost layer of the epidermis, composed of keratin‐filled corneocytes embedded in a lipid matrix, with a thickness ranging from 15 to 20 µm [2]. Despite its micrometer‐level thickness, the SC acts as the primary and stringent barrier to the penetration of exogenous substances into the skin [3]. Therefore, it is essential to determine the precise rate of active ingredient penetration through the SC barrier to understand the extent to which active ingredients are absorbed or utilized by the skin.

Currently, the most predominant techniques for penetration evaluation are Franz diffusion cell and tape stripping [4]. The Franz diffusion cell method stands as the primary approach for investigating skin penetration under ex vivo conditions [5]. It enables precise measurement of penetration through both the SC and deeper skin layers, employing techniques such as segmentation of the incubated skin [6]. Moreover, this method facilitates the measurement of permeation, indicating the diffusion of substances through the skin into a receptor medium. Tape stripping is a commonly employed minimally invasive technique, involving the application of adhesive tape onto the skin surface with a specified weight for a predetermined duration [7]. Subsequently, the tape is carefully removed, and the active substances are extracted from the keratinocyte layers adhering to the tape [8]. Typically, 10–20 strips are required to cover the entire SC depending on the thickness of the SC. Cyanoacrylate stripping, another low‐invasive method, can also be used to collect skin samples. Unlike tape stripping, which is primarily applied to the SC, cyanoacrylate stripping is applicable to both the SC and the viable epidermis, allowing for deeper penetration into the skin layers. Despite their widespread use, these methods are labor‐intense and time‐consuming, significantly impeding the continuous measurement of substance penetration [9].

In recent years, optical methods, with their non‐invasive nature, have become increasingly favored for enabling continuous measurements [10, 11]. Specifically, confocal Raman microspectroscopy (CRM) has stand out as a powerful technique due to its robustness, non‐invasiveness without the need for fluorescent markers. It is based on inelastic photon scattering to detect molecular vibrational modes, with CRM utilizing Stokes scattering, which provides higher intensity than anti‐Stokes scattering [12, 13]. The Raman intensity in the sample correlates linearly with the concentration of the substance, facilitating a direct correlation between substance concentration and Raman signal intensity [14]. CRM facilitates the creation of high‐resolution skin depth profiles and chemical maps, allowing for the visualization of spatial substance distribution and the quantification of concentration profiles across different skin layers [15].

Moreover, CRM's non‐invasive characteristics preserve the integrity of skin samples, allowing for longitudinal studies to monitor changes in ingredient penetration over time [16]. Notably, it uses red and near‐infrared wavelengths that penetrate skin with minimal fluorescence, making the Raman spectra clear and easy to interpret. Recent studies have extended CRM's applications by incorporating advanced chemometric methods to analyze Raman spectra more comprehensively. These methods, such as Partial Least Squares Regression (PLSR), Non‐Negative Matrix Factorization (NNMF), and Tailored Multivariate Curve Resolution‐Alternating Least Squares (tMCR‐ALS), are used to extract detailed penetration profiles of substances into skin, even when their Raman spectra overlap with skin components. These advancements enable more accurate determination of formulation penetration, stability, and metabolization within the skin.

Yeast/rice fermentation filtrate (RFF) is a fermentative extract enriched with small molecules, including amino acids, ceramides, organic acids, and vitamins, which are readily absorbed by the skin. Additionally, RFF contains larger bioactive compounds, such as polysaccharides and peptides, which offer notable skin care benefits. Studies have demonstrated that RFF can improve skin barrier function, enhance skin hydration, and reduce photoaging effects, particularly in human clinical trials [17, 18]. These properties make RFF a valuable ingredient in the formulation of moisturizing and anti‐aging cosmetics. Although several studies have shown that RFF can readily penetrate the skin, its penetration dynamics within the skin have been rarely investigated due to its complex composition [19, 20].

Herein, in this study, we aimed to study the in vivo penetration behavior of RFF on human skin by employing CRM technique. The penetration of RFF at multiple skin depths (10, 20, 30, and 40 µm) and time points (0.5, 1, 2, and 4 h) was accessed. Multivariate analysis was utilized to generate Raman spectral maps, illustrating the depth and spatial distribution of RFF across different skin layers.

## 2 Materials and Methods

### 2.1 Materials

RFF was provided by Mageline Biology Tech Co., Ltd. The provided RFF was prepared as follows: Saccharomyces yeast was first inoculated into a culture medium and activated at 30–40°C for 24 h to obtain a pre‐culture. Subsequently, pure water was added to rice, and the mixture was heated to 90–120°C for liquefaction. After cooling, amylase was introduced, and the saccharification reaction was allowed to proceed at 50–70°C for 48 h. The pre‐culture was then added to the saccharified rice mixture, and fermentation was carried out at 30–40°C. Finally, the fermentation filtrate was subjected to heat treatment at 90–120°C for sterilization, followed by cooling and filtration to yield the final rice fermentation filtrate. The water used in this experiment was ultrapure water.

### 2.2 Instrumentation

The Raman spectra were obtained by a high‐speed and high‐resolution confocal Raman microscope (LabRAM Odyssey, Horiba) with 532 nm laser excitation. The laser power used in the experiment was 2.68 mW, with an integration time of 0.5 s per point. The spectral acquisitions were recorded on the 400—4000 cm^−1^ spectral range.

### 2.3 CRM In Vivo

#### 2.3.1 Subjects

This nonmedical study was carried out on healthy human subjects in accordance with the Declaration of Helsinki, Good Clinical Practice (GCP) guidelines. The study was approved by the local ethic committee (ethic approval SHCPCH220604960‐1). Participants were informed both orally and in writing about the study's details, including any potential risks and inconveniences. A total of three healthy subjects (two males and one female, aged 20–25 years) were enrolled. Prior to their inclusion, all subjects provided their written consent. All participants successfully participated and completed the study.

#### 2.3.2 Climatic Conditions

Before the measurements, all subjects were instructed to cleanse the skin on the designated area of the volar forearm with clean water and stay in an air‐conditioned waiting area for 30 min (22 ± 2°C and at 50 ± 10% relative humidity). After 30 min of acclimatization, measurements were started in an airconditioned examination room with the same temperature and relative humidity.

#### 2.3.3 Procedure of In Vivo CRM Measurements

For in vivo measurements using CRM, we employed the inner forearm skin of a human subject. The forearm was positioned under the microscope objective, and the skin surface was carefully aligned and monitored using a 5× objective lens to ensure accurate positioning. The location was confirmed by observing the surface of the skin, focusing on the slight light‐dark fluctuations of the skin. To accurately determine the skin surface position, we used a higher‐order polynomial fitting method to calculate the point where the skin signal reaches half of its maximum value, which corresponds to the actual skin–air interface, or the outermost layer of the skin as previously described [21].

Raman spectra were obtained starting at the skin surface at 0 µm and going down to a skin depth of 40 µm with steps of 10 µm. Fifteen profiles were acquired with an integration time of 0.5 s and then averaged. Repeated measurements were recorded on different spots in a test area of 2 cm × 2 cm on the volar forearm. A point‐by‐point scanning mode was employed, starting from the skin surface and moving through the skin–air interface down to the deeper layers of the skin. Raman spectra were obtained at different spatial points, which were subsequently processed and analyzed.

Depth Raman imaging was conducted in the X‐Z direction using a point‐by‐point scanning method. The longitudinal step interval for spectral data collection was 5 µm, with a scanning area of 20 µm × 200 µm. Each imaging session covered a 20 × 200 µm area with 205 points acquired, taking approximately 102 s. Including grating movement and optical path calibration, the total measurement time was typically 120–150 s. This allows for precise data acquisition within the defined scanning area.

### 2.4 Data Processing

Raman spectral imaging data processing involved several steps, including spectrum preprocessing and data analysis. Spectrum preprocessing tasks included cosmic ray removal, spectrum smoothing, background noise elimination, baseline calibration, and spectrum normalization. Univariate data analysis was performed to identify the presence of biochemical substances in the skin based on specific spectral peaks. Background signal subtraction was performed using multivariate signal correction according to a previous study [21].

Firstly, the obtained Raman spectra were preprocessed. A multi‐step nonlinear dimensionality reduction algorithm was then employed to compress the Raman spectral information at various skin depths. The boundary between the SC and the viable epidermis was determined based on the distinct morphological differences and biological markers between these layers [22, 23, 24]. The SC has lower water content and is characterized by a high degree of order in keratin and lipids. We defined the boundary between the SC and viable epidermis as the point where the curves of keratin/lipid ordering and SC water content intersect. Specifically, the boundary between the SC and viable epidermis is determined by the intersection of the amide I band intensity curve with the water‐related OH band intensity curve. The depth at which the sum intensity of the keratin‐associated amide I band reaches its maximum and minimum values is used to define this boundary. For data analysis, we applied a seventh‐order polynomial to process the normalized spectral data. Depth‐dependent Raman spectra and normalized intensity curves of specific peaks show that the water‐related OH peaks steadily increase with depth. In contrast, the intensity of keratin‐ and lipid‐related peaks initially increases and then decreases after reaching the interface between the SC and viable epidermis. These peak intensity changes allow us to accurately calculate the thickness of the SC and the boundary between the SC and viable epidermis. To differentiate the dermis from the epidermis, we examined the structural boundary at the dermoepidermal junction, where desmosomes are exclusively localized [25, 26]. These specialized protein structures, absent in the epidermis, serve as a unique marker for identifying the dermal–epidermal interface (Figure S1). This boundary was used in our study to delineate the two skin layers for further analysis.

Additionally, the data were processed using the subtraction method and multivariate curve resolution‐alternating least squares (MCR‐ALS) regression method to isolate the Raman characteristic information. The spatial distribution of these characteristic Raman signals was then utilized to reconstruct a two‐dimensional false‐color map. For data analysis, Labspec5 software was used to calibrate the baseline of Raman spectra and identify characteristic peaks. Various parameters, including peak intensity, displacement, area, and half‐peak width, were computed. Labspec6 software also enabled numerical analysis of peak intensity across different depths and spatial distribution. By utilizing characteristic Raman signals distinguishing the sample from intrinsic skin signals, the permeation behavior of the sample across different skin layers was verified.

The relative permeability (RP) within each layer of human in vivo skin was calculated using the total RP value as 100%, distributing values across the SC, viable epidermis, and dermis within the test areas. The specific calculation formula is as follows (applied within the wavenumber range of 400–3200 cm⁻¹):

$$RP\;\left(\%\right)=\frac{\mathrm{Raman}\;\mathrm{spectral}\;\mathrm{intensity}\;\mathrm{of}\;\mathrm{RFF}\;\mathrm{within}\;\mathrm{the}\;s\mathrm{kin}}{\mathrm{Raman}\;\mathrm{spectral}\;\mathrm{intensity}\;\mathrm{of}\;\mathrm{RFF}\;\mathrm{applied}\;\mathrm{on}\;\mathrm{the}\;\mathrm{skin}\;\mathrm{surface}}\;\times 100\%$$

### 2.5 Statistical Analysis

Statistical analyses were performed using GraphPad Prism software. Data are presented as mean ± standard deviation (SD). Differences between the blank control group and experimental groups, as well as differences among experimental groups, were analyzed using a two‐tailed *t*‐test for single‐factor paired comparisons. A *p* value of less than 0.05 (*) was considered statistically significant.

## 3 Results

### 3.1 Spectral Signature and Features of RFF

**FIGURE 1:**
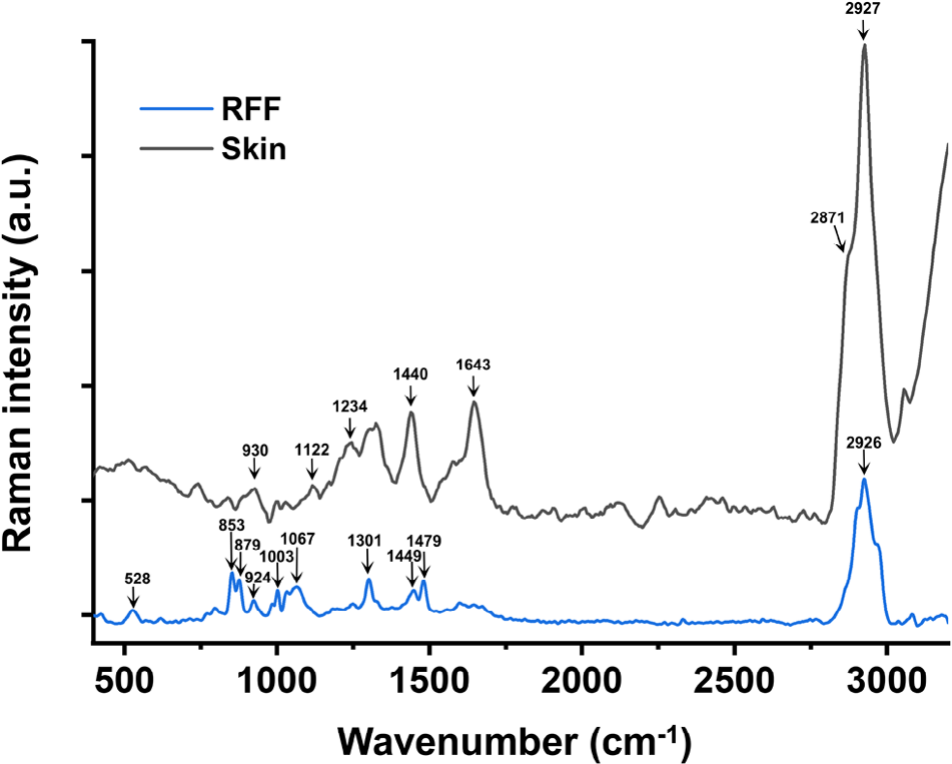
Raman spectrum of human forearm skin surface and RFF, showing distinct differences.

CRM was employed to characterize the Raman spectra of RFF to identify its characteristic vibrational bands. Figure 1 shows the Raman spectra of both skin and RFF in the range of 400–3200 cm^−1^. The characteristic Raman peaks of RFF appear at 528 cm⁻¹, 853 cm⁻¹, 879 cm⁻¹, 924 cm⁻¹, 1003 cm⁻¹, 1301 cm⁻¹, 1449 cm⁻¹, 1479 cm⁻¹, and 2926 cm⁻¹ (Table 1), demonstrating that CRM can effectively detect RFF signals despite its complex composition. In comparison, the skin exhibits distinct Raman peaks at 930 cm⁻¹, 1122 cm⁻¹, 1234 cm⁻¹, 1440 cm⁻¹, 1643 cm⁻¹, 2871 cm⁻¹, 2927 cm⁻¹, and 3059 cm⁻¹ (Table 2), underscoring the distinct Raman spectral profiles of RFF and skin.

**TABLE 1:** Assignment of Raman characteristic peaks of RFF and its corresponding representative components.

Peak position	Vibration mode	Main representative components
528 cm^−1^	S‐S disulfide stretching in proteins	Proteins
853, 879 cm^−1^	(C‐O‐C) backbone	Sugars
924 cm^−1^	C‐C stretching vibration	Proteins
1003, 1067 cm^−1^	C‐C backbone vibration	Lipids
1301 cm^−1^	CH₂ twisting and deformation	Proteins, sugars, lipids
1449, 1479 cm^−1^	CH₂ bending of amides and lipids	Proteins, lipids
2926 cm^−1^	C‐C cyclic backbone vibration	Lipids, amino acids

**TABLE 2:** Assignment of Raman characteristic peaks in the skin and their corresponding representative components.

Peak position	Vibration mode	Main representative components
930 cm⁻¹ 1122 cm⁻¹	N(C‐C) skeleton, collagen skeleton	Proline, hydroxyproline
1234 cm⁻¹	C‐N absorption band (Amide III band)	Glycine skeleton, proline, nucleic acids
1440 cm⁻¹	C‐H bending mode of protein (CH₂ stretching/CH₃ asymmetric deformation)	Structural proteins, elastin
1643 cm⁻¹	νC=O stretching vibration (Amide I band, including α‐helix, β‐sheet, and random coil)	Actin, collagen, keratin
2871 cm⁻¹ 2927 cm⁻¹	CH₂ asymmetric stretching	Lipids
3059 cm⁻¹	CH₂ symmetric stretching	Lipids

### 3.2 Raman Spectra of RFF‐Treated Skin and Background Interference Removal

**FIGURE 2:**
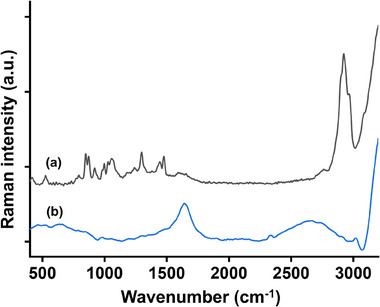
(a) Original Raman spectrum of the skin surface treated with RFF, and (b) the difference Raman spectrum of the RFF‐treated skin surface after removal of skin background interference and non‐negative constraint correction treatment.

Figure 2a shows the original Raman spectrum of the RFF‐treated skin, with peaks corresponding to both RFF and the skin. To minimize background interference from the skin, a background subtraction method was applied to the RFF and skin spectra, followed by non‐negative constraint correction. The refined spectrum, shown in Figure 2b, effectively represents the difference between the RFF and skin spectra, allowing for a more accurate evaluation of RFF penetration into the skin.

### 3.3 Temporal and Depth‐Dependent Analysis of RFF Permeability in Skin

**FIGURE 3:**
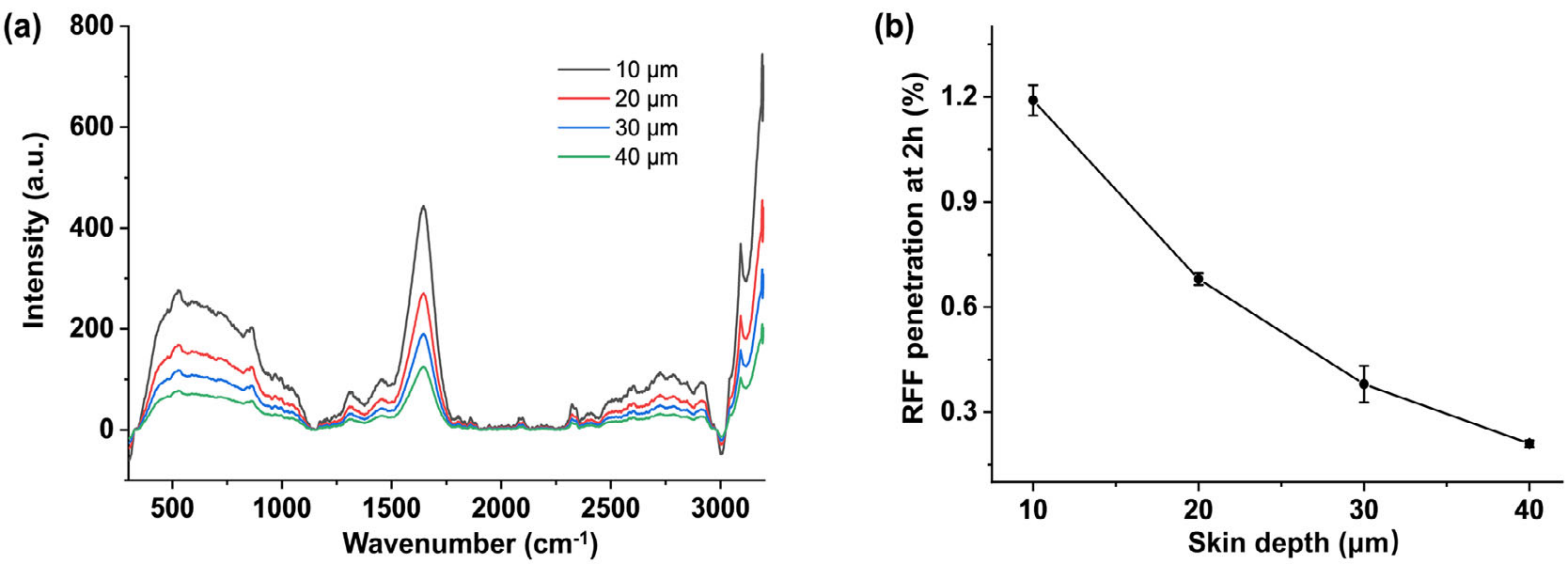
(a) Raman spectra of RFF‐treated skin after 2 h at skin depths of 10, 20, 30, and 40 µm. Background subtraction was applied during spectra preprocessing. (b) Quantification analysis showing the relative RFF permeability at different skin depths 2 h post‐application. The results are shown as mean ± standard deviation (*n* = 3).

CRM provided detailed insights into the temporal and depth‐dependent distribution of RFF within the skin. Figure 3a illustrates the Raman spectra of RFF at different skin depths after a 2‐h application period. The highest Raman signal was observed at a depth of 10 µm, gradually decreasing toward 40 µm, indicating a reduced concentration of RFF with deeper penetration into the skin. Quantitative analysis confirmed RFF permeability percentages of 1.19%, 0.68%, 0.38%, and 0.21% at depths of 10, 20, 30, and 40 µm, respectively (Figure 3b). These findings demonstrate the capability of CRM to accurately assess and quantify the distribution of RFF across different skin depths.

**FIGURE 4:**
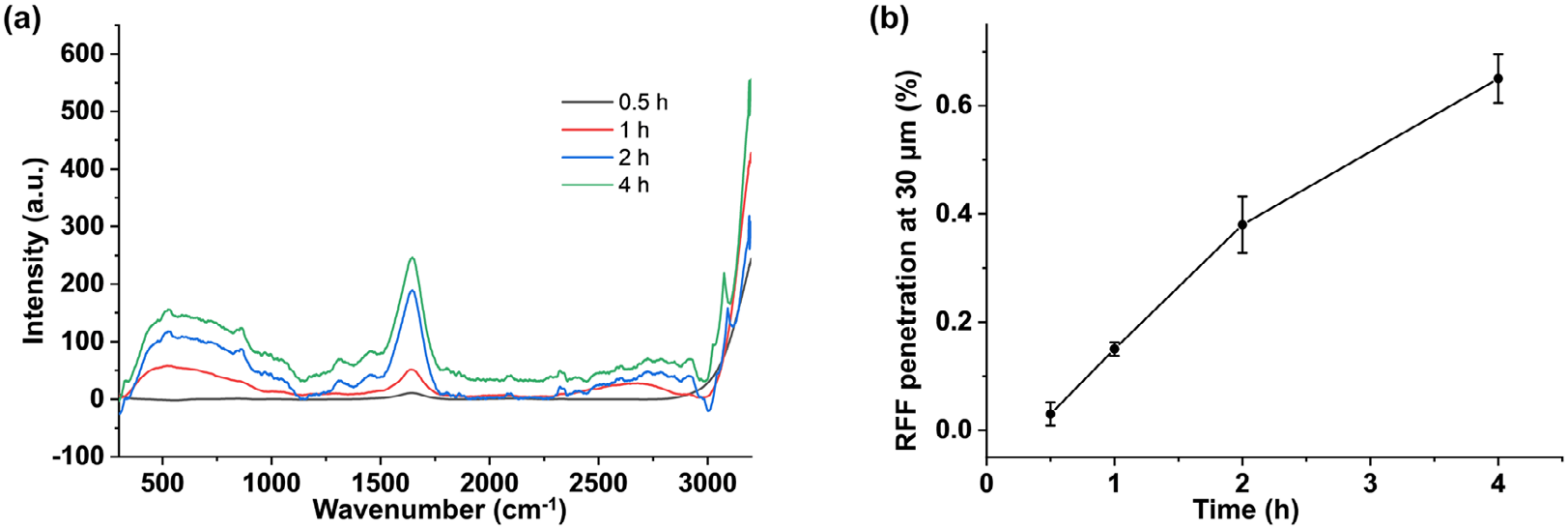
(a) Raman spectra of RFF‐treated skin at a depth of 30 µm at 0.5, 1, 2, and 4 h. Background subtraction was applied during spectra preprocessing. (b) Quantitative analysis showing the relative permeability at a skin depth of 30 µm across various time intervals. The results are shown as mean ± standard deviation (*n* =3).

Figure 4a shows the Raman spectra of RFF‐treated skin at a depth of 30 µm over various time intervals. These spectra illustrated increased RFF penetration with prolonged application time. RFF permeated the SC barrier within 30 min, demonstrating rapid kinetics of permeation. At 2 and 4 h of application, a more pronounced RFF signal was observed, indicating deeper penetration into the skin. Quantitative analysis of RFF levels, detailed in Figure 4b, shows permeation rates of 0.03%, 0.15%, 0.38%, and 0.65% at 0.5, 1, 2, and 4 h, respectively.

### 3.4 Measurement of RP of RFF Into the Skin

**FIGURE 5:**
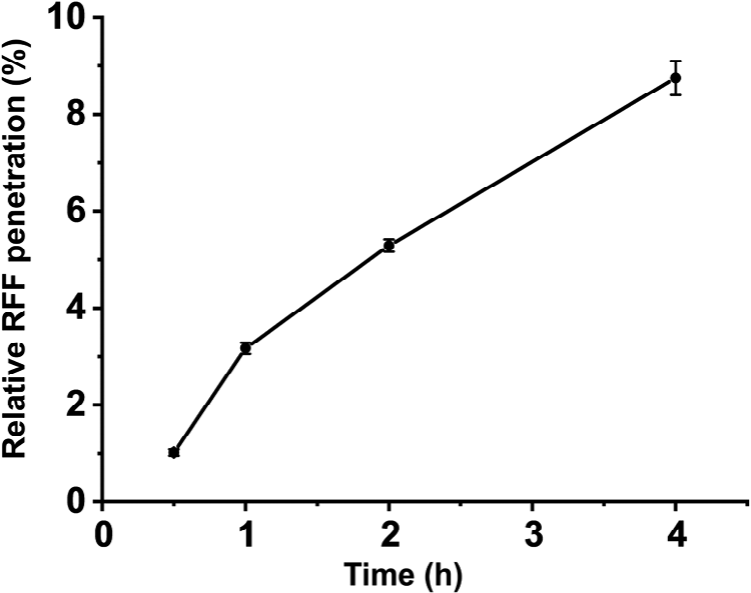
The Relative RFF permeability at 0.5, 1, 2, and 4 h post‐application. The results are shown as mean ± standard deviation (*n* = 3).

Figure 5 presents the RP of RFF into the skin, normalized by the change in Raman characteristic peaks induced by RFF post‐application. Within 0.5 h of application, RFF permeability into the skin was measurable at 1.02%. As the application time increased to 1 h, RFF permeation increased to 3.17%, demonstrating a steady increase in absorption over time. Continued application for 2 and 4 h resulted in further increases in RFF permeation, reaching 5.29% and 8.75%, respectively. These results highlighted a time‐dependent increase in RFF permeability, with significant increases observed at each successive time interval.

### 3.5 Spatial Distribution of RFF Within the Skin

**FIGURE 6:**
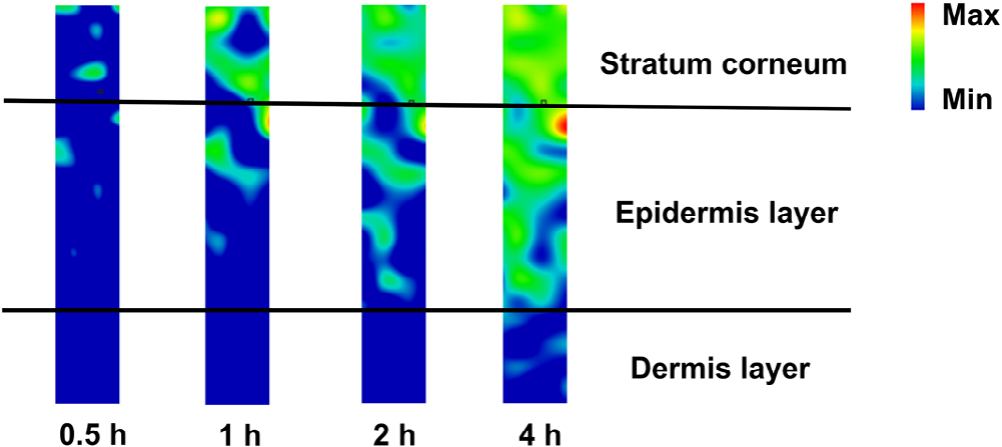
Heat map illustrating the spatial distribution of RFF concentration across different skin layers after varying application durations.

Combined with multivariate analysis, the Raman spectral map enabled the calculation of RFF's abundance fraction at each time point (Figure 6). The resulting heat map illustrated the concentration distribution of RFF, clearly showing a gradual increase in its penetration into the skin over the 4‐h application period. Initially, within 0.5 h, RFF entered the SC and began permeating the viable epidermis. Penetration through both layers increased by 1 and 2 h, with higher concentrations observed. By 4 h, a significant amount of RFF had penetrated into the epidermis layer, with some reaching the dermis layer.

## 4 Discussion

The present study demonstrates the utility of CRM in evaluating the in vivo penetration of RFF. Traditional methods such as the Franz diffusion cell and tape stripping rely on subsequent HPLC and other quantitative techniques to characterize substance amount, which can be inefficient for complex mixtures like RFF [27, 28, 29]. Raman spectroscopy offers significant advantages for detecting and localizing exogenous molecules with micrometric resolution in a label‐free, non‐destructive manner with minimal sample preparation [30, 31, 32]. Currently, there is a paucity of published data on the penetration depth of RFF into the skin, and to our knowledge, no studies have directly addressed this issue. This gap in the literature highlights the significance of our study, as the complex nature of RFF, with its diverse constituents, poses challenges in assessing its skin penetration. Despite being a complex mixture, we found that RFF has a relatively simple Raman spectrum, characterized by a few distinct peaks. Notably, the Raman spectrum of RFF on the skin differs significantly from that of the skin, enabling CRM to effectively elucidate the penetration dynamics of RFF.

In this pilot study, we demonstrated that RFF surpassed the SC barrier (>20 µm) within just 30 min of application, indicating rapid permeation kinetics. Quantitative analysis confirmed increasing RFF penetration into the skin over time, with levels reaching 8.75% after 4 h. Notably, the variability among the three subjects was negligible, indicating strong reproducibility of the Raman measurements. This consistency is likely due to CRM's reliance on the inherent properties of RFF. This method allows for detection without labeling or processing, thereby accurately reflecting the penetration behavior of RFF in the skin.

Moreover, CRM can provide advanced information on RFF penetration and permeation, such as skin depth scanning and visualized spatial concentration distribution. By tracking the characteristic peak of RFF at 1642 cm⁻¹, penetration into skin depths from 10 to 40 µm was achieved within 2 h. The results showed that the Raman signal of RFF diminished with increasing depth into the skin, demonstrating CRM's capability to accurately measure and quantify the presence of RFF at various depths. Coupled with multivariate analysis, Raman spectral maps enable the tracking of RFF depth penetration and its spatial distribution across different skin layers. The concentration distribution heat map revealed RFF's ability to enter the dermal layers, with a notable increase observed with extended application durations. This visualized spatial distribution underscores the depth and concentration of RFF within the skin, providing comprehensive insights into its permeation behavior.

Despite these promising results, this pilot study has some limitations. Measurements were conducted on a relatively small sample size of three subjects and on volar forearm skin, potentially limiting the generalizability of the findings. The small sample size might not fully capture the variability in penetration dynamics across a broader population. However, the robustness of CRM in detecting and quantifying RFF, even within complex mixtures, underscores its potential applicability to various substances. This method's strong reproducibility and effectiveness suggest that CRM could be widely adopted for assessing the penetration of RFF and other fermented ingredients.

## 5 Conclusion

In conclusion, this work demonstrates that CRM is a powerful and effective tool for evaluating the in vivo penetration of RFF into human skin. The CRM results indicated an increasing penetration of RFF with extended application duration, reaching 1.02% and 8.75% after 0.5 and 4 h, respectively. Additionally, the concentration distribution heat map revealed that RFF surpassed the SC barrier within 30 min and reached the dermis layer after 4 h of application, indicating rapid permeation kinetics. This study presents a promising approach for evaluating the penetration of complex compounds with measurable Raman peaks, such as RFF, using CRM, and this method can be extended to other active substances.

## Conflicts of Interest

The authors declare no conflicts of interest.

## Supporting information



Supporting Information

## Data Availability

The data that support the findings of this study are available on request from the corresponding author.
