# Supplementary figures and images for: Evaluating In Vivo Penetration of Yeast Rice Ferment Filtrate Into Skin Using Confocal Raman Microspectroscopy: A Pilot Study
Source: Skin Res Technol. 2025 May 5;31(2-5):e70166. doi: 10.1111/srt.70166 (PMC12050641; doi:10.1111/srt.70166)

**Figure S1.** Raman spectrum of desmosomes.


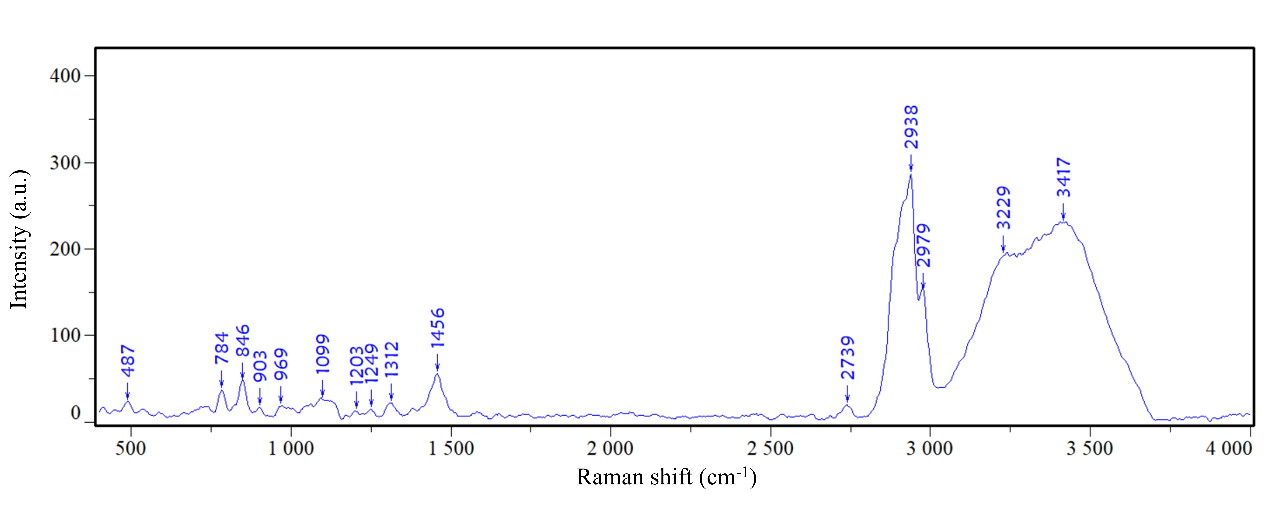

Supplement: Supplementary file 1 — Supporting Information [file SRT-31-e70166-s001.docx]
